# Formation and fragmentation of doubly and triply charged ions in the negative ion spectra of neutral *N*-glycans from viral and other glycoproteins

**DOI:** 10.1007/s00216-021-03480-8

**Published:** 2021-08-03

**Authors:** David J. Harvey, Weston B. Struwe, Anna-Janina Behrens, Snezana Vasiljevic, Max Crispin

**Affiliations:** 1 Present Address: Oxford Glycobiology Institute, Department of Biochemistry, University of Oxford, South Parks Road, Oxford, OX1 3QU UK; 2 Target Discovery Institute, Nuffield Department of Medicine, University of Oxford, Roosevelt Drive, Oxford, OX3 7FZ UK; 3 Chemistry Research Laboratory, Department of Chemistry, University of Oxford, South Parks Road, Oxford, OX1 3TA UK; 4 Present Address: GlycoEra AG, Grabenstrasse 3, 8952 Schlieren, Switzerland; 5 School of Biological Sciences, Faculty of Natural and Environmental Sciences, University of Southampton, Highfield Campus, Southampton, SO17 1BJ UK

**Keywords:** *N*-Glycans, Double charge, Triple charge, Negative ion, Fragmentation

## Abstract

Structural determination of *N*-glycans by mass spectrometry is ideally performed by negative ion collision-induced dissociation because the spectra are dominated by cross-ring fragments leading to ions that reveal structural details not available by many other methods. Most glycans form [M – H]^-^ or [M + adduct]^-^ ions but larger ones (above approx. *m/z* 2000) typically form doubly charged ions. Differences have been reported between the fragmentation of singly and doubly charged ions but a detailed comparison does not appear to have been reported. In addition to [M + adduct]^-^ ions (this paper uses phosphate as the adduct) other doubly, triply, and quadruply charged ions of composition [M_n_ + (H_2_PO_4_)_n_]^n^^-^ have been observed in mixtures of *N*-glycans released from viral and other glycoproteins. This paper explores the formation and fragmentation of these different types of multiply charged ions with particular reference to the presence of diagnostic fragments in the CID spectra and comments on how these ions can be used to characterize these glycans.

**Graphical abstract:**

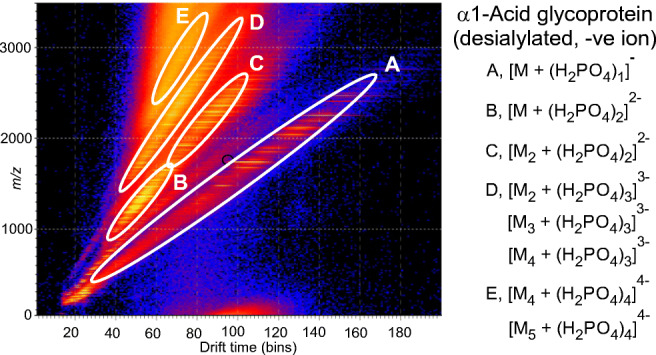

## Introduction

**Scheme 1:**
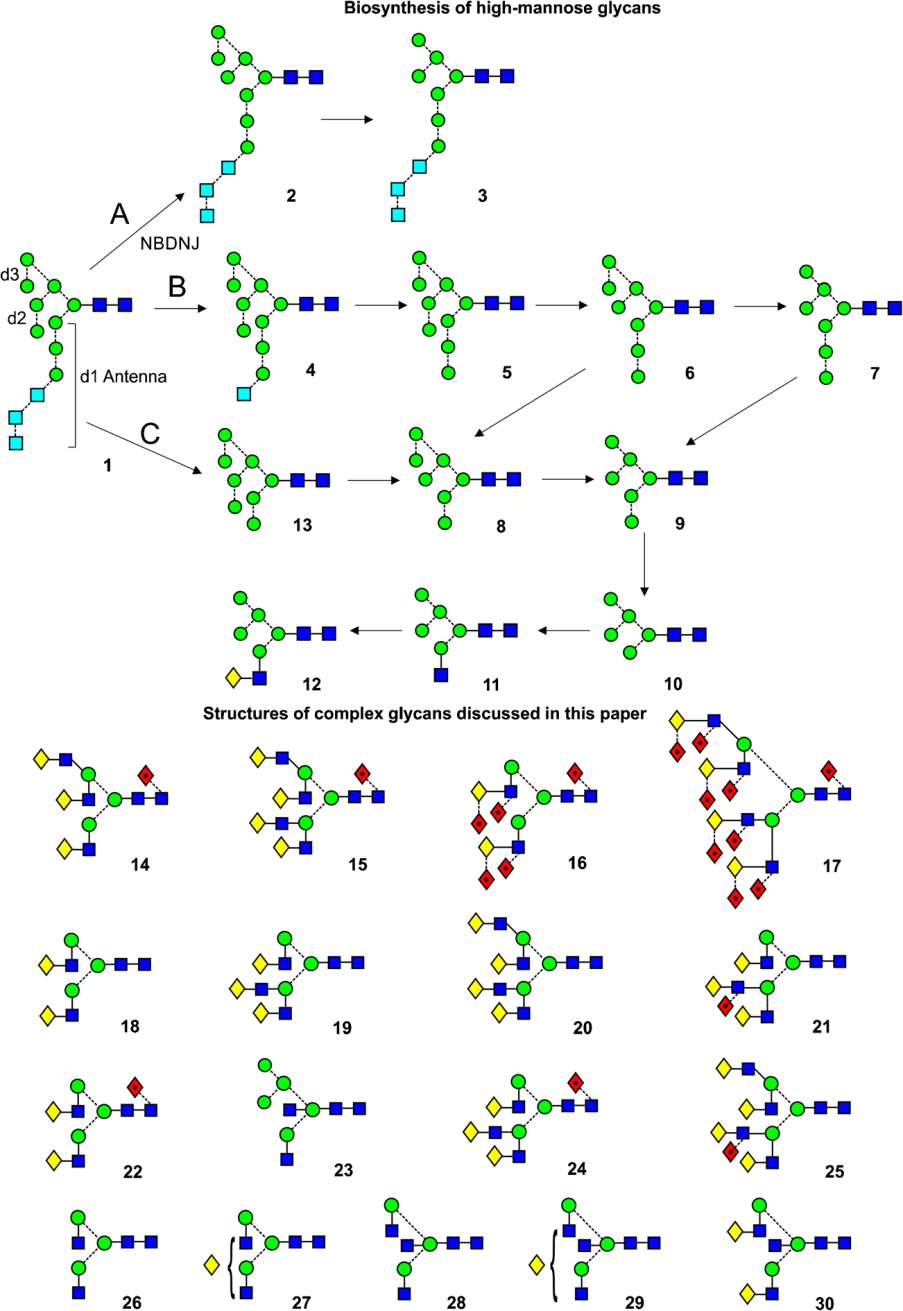
**T**op, biosynthesis of high-mannose glycans. Glycan **1** is attached to the protein at the GlcNAc terminus. Path A, If glucose removal is blocked with drugs such as NBDNJ, then only the outer mannose residues are removed (glycans **2** and **3**) by the enzymes of the normal pathway (Path B, glycans **4**–**12**). Pathway C operates under these conditions with use of an endomannosidase to produce an isomer of Man_8_GlcNAc_2_ (**13**) which then enters the normal pathway. The two outer mannose residues are removed from glycan **11** leading to the synthesis of complex glycans such as glycans **14** – **30** that are discussed in this paper. The galactose residues of the hybrid (**12**) and complex glycans are frequently capped with sialic acid. Symbols used for the glycans are  = mannose,  = GlcNAc,  = glucose,  = fucose, and  = galactose. Solid lines connecting the symbols are β-linkages; broken lines are α-linkages. The angle of the lines shows the linkage position. For more information see [2].

*N*-Linked glycans are those attached to proteins in an Asn-Xxx-Ser/Thr motif where X is any amino acid except proline. They play essential roles in areas such as cell-cell recognition, glycoprotein folding, protection from immune recognition, fertilization, and reproduction [1]. Broadly speaking, these compounds can be divided into three types (Scheme 1) depending on the glycan chains that are attached to the common trimannosyl-*N,N*’-di-acetyl-chitobiose core: High mannose glycans **1**-**10** and **13** (Scheme 1) where three chains consist of mannose residues, sometimes with one capped with up to three glucose residues (**1**-**4**), hybrid glycans such as **11** and **12** that contain a mixture of mannose and GlcNAc-Gal (or GalNAc) chains and complex glycans (e.g., **14**-**30**) where all mannose residues, except those in the core, are replaced with similar GlcNAc-Gal chains. Many of these chains can be capped with acid groups such as *N*-acetyl-(or *N*-glycolyl)-neuraminic acid, sulfate, or phosphate groups or can be extended by the addition of additional Gal-GlcNAc moieties.

Many methods have been used to determine the structures of these glycans (see, for example, [3–13]). Early studies by mass spectrometry relied on combined gas chromatography/mass spectrometry (GC/MS) and fast atom bombardment (FAB) [14] ionization but most studies now use matrix-assisted laser desorption/ionization (MALDI, see Harvey [15] and reviews cited therein) or electrospray ionization (ESI), the latter method being conveniently interfaced with high-performance liquid chromatography (HPLC). A potential problem with ESI is the preferential production of ions in several charge states, thus inhibiting acquisition of a quantitative glycan profile from mixtures. MALDI produces essentially only singly charged ions allowing better profiling of mixtures but suffers from the disadvantage that sialylated glycans tend to eliminate sialic acids. This latter problem, however, can be overcome by permethylation or better, ester [16] or amide formation from the acid groups of the sialic acids. This latter derivatization method has been developed into a linkage-specific technique [17–19] and incorporated into many HPLC/MS analyses.

Although positive ion fragmentation mass spectrometry, used by most of the above techniques, provides much structural information, better information can be obtained by negative ion methods [20]. Neutral glycans (those not containing acid groups such as Neu5Ac) are best examined with tandem instruments by forming adducts of the type [M + A_n_]^n-^ (where A is the anion) with anions such as chloride, nitrate, or phosphate, in order to inhibit fragmentation in the mass spectrometer ion source. Their fragmentation spectra, usually acquired by collision-induced dissociation (CID), although generally containing fewer fragments that their positive ion counterparts, are dominated by cross-ring fragments that provide specific information on, for example, location of fucose residues and the presence or absence of bisecting GlcNAc residues that is often difficult to determine by traditional positive ion methods or by exoglycosidase digestions. Another advantage of negative ion fragmentation is that isomeric glycans usually fragment to give mass-different ions rather than the predominantly abundance-different ions commonly seen in positive ion spectra. Consequently, their presence is usually obvious from the CID spectra.

Deprotonated ions can also be formed under ESI conditions but there is a tendency for double charging ([M – H_2_]^2-^ ions). Fragmentation of these doubly charged ions often differs somewhat from those of the [M – H]^-^ [21] and phosphate adducts even though the first stage of the fragmentation of the latter ions is to deprotonate the molecular ion. Reasons for this difference have been discussed but without a satisfactory conclusion mainly because of differences in the experimental conditions (collision gas, collision energy, etc.) that prevent a strict comparison [22]. Sialylated glycans (not derivatized) under ESI conditions tend to deprotonate rather than form adducts but their fragmentation spectra are less informative than those of the neutral glycans because of domination by fragment ions formed by charge localization of the acid group rather than on a hydroxyl group, as is the case with neutral glycans. The diagnostic ions seen from adducted neutral glycans are generally missing but can be restored by derivatization as described above.

The recent introduction of ion mobility to commercial instruments has provided another dimension to the analysis of these glycans [23–25]. Although resolution is currently inferior to that provided by HPLC, the technique is much more rapid (ms time scale) and provides the ability to separate ions into groups of different charge states and, because of its sensitivity to molecular shape, to enable separation of some isomers, a property not directly available to mass spectrometry. The physical property associated with ion mobility is a molecule’s collisional cross section which is instrument independent and provides another parameter for compound identification. Together with negative ion fragmentation, this combined technique provides one of the most powerful analytical method for *N*-glycan analysis available today. When examined by ESI, many glycans, particularly the larger ones, produce doubly and triply charged ions and the ability of ion mobility to separate glycans on the basis of charge is used here to extract these ions from mixtures and to allow many of the minor ions, often hidden by “noise” and not seen before, to be identified.

One of the applications of ion mobility/negative ion CID technique employed in this laboratory is the structural identification of *N*-glycans from viruses. Enveloped viruses, such as the human immunodeficiency virus (HIV), contain heavily glycosylated spike proteins in their surface layer, or spike as is the case with COVID-19, and are possible targets for vaccine development [26]. Negative ion fragmentation, usually combined with ion mobility, has been applied to glycoproteins from a number of viruses such as influenza (hemagglutinin and neuraminidase) [27], Ebola (transmembrane glycoprotein (GP1) and the soluble glycoprotein (sGP) [28]), SARS (spike glycoprotein [29]), HIV (gp120 and gp41 glycoproteins [30–37]), Nipah virus [38], Hendra virus attachment glycoprotein [39], Machupo virus attachment protein [40], swine fever virus (E2 glycoprotein [41]), Lassa virus [42], Uukuniemi phlebovirus [43], and Semliki Forest virus (E1 and E2 glycoproteins [44]), mainly of recombinant origin from systems such as human embryonic kidney (HEK) 293 and Chinese hamster ovary (CHO) cells. These studies have shown the occurrence of high-mannose, hybrid, and complex glycans with high-mannose glycans being particularly abundant in the heavily glycosylated glycoproteins such as gp120 from HIV [33]. Both singly and multiple-charged ions are produced with some of the doubly charged ions from gp120 being of a type not observed previously from negative ions.

Because as yet, there does not appear to have been a systematic comparison of the formation and fragmentation of negative ions produced with multiple charge states, this paper utilizes the power of ion mobility to extract these ions and discusses the formation, fragmentation, and uses of them. In addition to the common [M + A_n_]^n-^ and [M – H_n_]^n-^ ions, it also reports other novel types of multiply charged ions that can be formed and which have been revealed by ion mobility.

## Methods

### Materials

Reference glycans were purchased from Dextra Laboratories (Reading, UK). Glycans from HIV gp120 and gp41 were released in-gel with peptide *N*-glycosidase F (PNGase F from New England Biolabs (UK), Hitchin, UK) [45] as described earlier [33]. Fucosylated glycans from human parotid gland glycoproteins, obtained from banked deidentified human tissue, were released with hydrazine and re-acetylated, also as described earlier [46]. Human α1-acid glycoprotein (AGP) was obtained from Oxford GlycoSystems (Abingdon, UK). Glycans were released by PNGase F and desialylated by heating with 1 M acetic acid for 10 min at 80°C.

### Mass spectrometry

#### [M + adduct ions]

Released glycans in 2 μL of water were cleaned with a Nafion membrane [47] prior to analysis. All glycans were then dispersed into 1:1 (v:v) water:methanol (~6 μL) to which 0.2 mL of an 0.5 mM solution of ammonium phosphate had been added (to form phosphate adducts of the glycans). Travelling wave ion mobility mass spectrometry (TWIMS) measurements were performed with a Synapt G2Si travelling wave ion mobility mass spectrometer (Waters, Manchester, UK) [48] fitted with a nano-ESI (nESI) ion source. Gold-coated borosilicate capillaries, prepared in-house [49], were used for introducing the samples. Infusions lasted from 2 to 3 h (50–33 nL/min). Ion source conditions were as follows: ESI capillary voltage, 1.0–1.2 kV cone voltage, 100–180 V, ion source temperature 80°C. The T-wave velocity and peak height voltages were 450 m/s and 40 V respectively with nitrogen in the TWIMS cell. Fragmentation was performed after mobility separation in the transfer cell with argon as the collision gas. The collision cell voltage (60–130 V) was adjusted manually according to the precursor ion mass to give an even distribution of fragment ions across the mass range. The instrument was externally mass calibrated with dextran oligomers (Glc_2-13_) from *Leuconostoc mesenteroides*. Data acquisition and processing were carried out using the Waters DriftScope (version 2.8) software and MassLynx^TM^ (version 4.1). The scheme devised by Domon and Costello [50] was used to name the fragment ions with the addition that the subscript R sometimes replaced the numerical subscript on the reducing-terminal GlcNAc residue in the following discussion to avoid the number changing with glycans with different chain lengths. R-1 replaced the corresponding subscripts for the penultimate GlcNAc residue and for the B ion separating them. Interpretation of the fragmentation data followed the rules established earlier [51–55].

#### [M – H_2_]^2-^ ions

Released glycans were cleaned as above but spectra were recorded mainly with a Waters Ultima Global Q-TOF instrument. Where fragmentation spectra were recorded on both instruments, they were identical. Samples (~50 pmol/L) in 1:1 (vol:vol) methanol:water containing 0.1 M ammonium hydroxide were infused at 5 μL/min with a diffusion pump and with a potential of 3.0 kV on the ESI needle. The ion source was maintained at 120°C, the nebulizer gas was 100°C, and the cone and desolvation nitrogen flows were 50 and 450 L/h, respectively. The cone voltage was 100 V and the RF-1 voltage was set at 180 and 80 V for singly and doubly charged ions, respectively. Spectra (2-s scans) were acquired with a digitization rate of 4 GHz and accumulated until a satisfactory signal:noise ratio had been obtained (noise level less than about 1%). For MS/MS data acquisition, the precursor ion was selected at low resolution (5 *m/z* mass window) to allow transmission of isotope peaks and fragmented with argon at a pressure (recorded on the instrument’s pressure gauge) of 0.5 bar. The voltage on the collision cell was adjusted with mass and charge to optimize formation of the diagnostic fragment ions. Typical values were 80–120 V for the singly charged ions and 30–50 V for the doubly charged ions. Other voltages were as recommended by the manufacturer. Instrument control and data acquisition were performed with a MassLynx data system (Version 4.0) with processing as above.

## Results and discussion

### Formation of multiply charged ions

#### Ions of type [M – H_n_]^n-^ and [M + (H_2_PO_4_)_n_]^n-^ from gp120

**Fig. 1:**
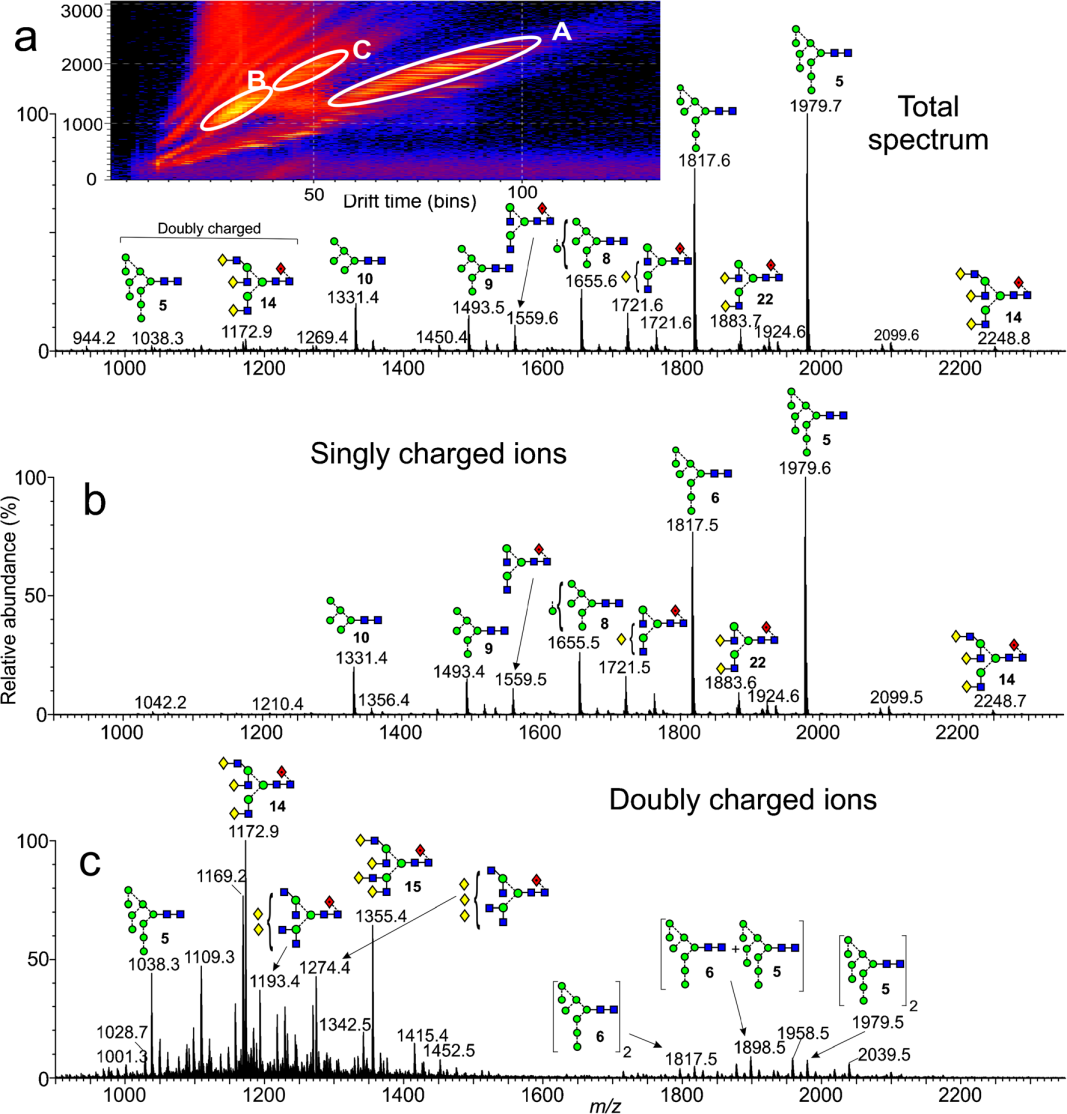
Mobility extracted singly ([M + H_2_PO_4_]^-^) and doubly ([M + (H_2_PO_4_)_2_]^2-^) negatively charged ions from *N*-glycans released from HIV gp120 (SOSIP from HEK293F cells) recorded with a cone voltage of 150 V. (**a**) Total spectrum. The inset shows the DriftScope (drift time:*m/z*) profile (A = singly charged ions, B = doubly charged ions of composition [M + (H_2_PO_4_)_2_]^2-^, and C = doubly charged ions of composition [M_2_ + (H_2_PO_4_)_2_]^2-^). (**b**) Mobility extracted singly charged ions. (**c**) Mobility extracted doubly charged ions. Symbols for the glycan structures in this and later figures are as defined in the legend to Scheme 1

*N*-Glycans naturally form deprotonated ions under negative ESI conditions with both [M – H]^-^ and [M -H_2_]^2-^ ions being produced. In the presence of phosphate, the major ions from neutral glycans are [M + H_2_PO_4_]^-^ or [M + (H_2_PO_4_)_2_]^2-^. Doubly charged ions and ions in higher charge states are preferentially produced from larger glycans in a mixture (e.g., [56]), probably because the charges can be better distributed, thus reducing Coulombic repulsion [57]. Figure 1c shows the preferential formation of doubly charged ions of the type [M + (H_2_PO_4_)_2_]^2-^ from the larger high-mannose glycans from gp120 produced in HEK293F cells and recorded with a Waters Synapt G2Si instrument with a relatively high cone voltage. Mobility-extracted singly charged ions from neutral glycans are shown in Fig. 1b and those from the doubly charged ions are displayed in Fig. 1c. In addition to the presence of doubly charged high-mannose glycans, Fig. 1c reveals prominent ions from the fucosylated triantennary glycan (**14**, *m/z* 1172.9), which is about three times as abundant as the corresponding singly charged ion (*m/z* 2248.8). The tetra-antennary glycan (**15**) also produced a prominent doubly charged ion at *m/z* 1355.4 but the singly charged ion is hardly detectable emphasizing the need to investigate ions in both charge states to deduce the total glycan profile.

#### Ions of type [M_2_ + (H_2_PO_4_)_n_]^n-^ from gp120

A second set of doubly charged ions having the composition [M_2_ + (H_2_PO_4_)_2_]^2-^ in the *m/z* 1700 – 2200 region appeared in the spectrum of gp120 glycans (Fig. 1c). Although both high-mannose and some complex tri- and tetra-antennary glycans were found in this sample, only the high-mannose glycans were observed to form [M_2_ + (H_2_PO_4_)_2_]^2-^ ions (Table 1).

**Table 1:**
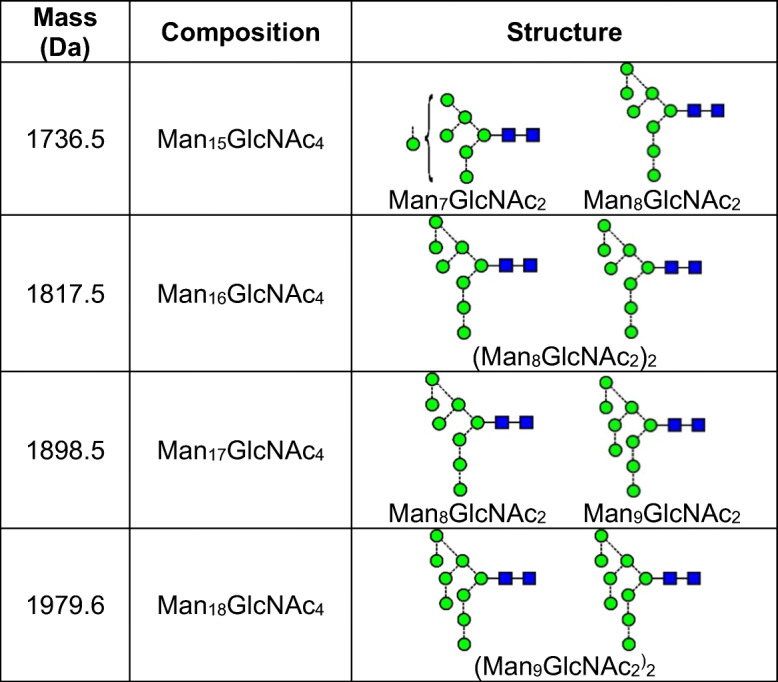
Compositions of the dimeric ions of composition [M + (H_2_PO_4_)_2_]^2-^ found in the spectrum of *N*-glycans from HIV gp120

**Fig. 2:**
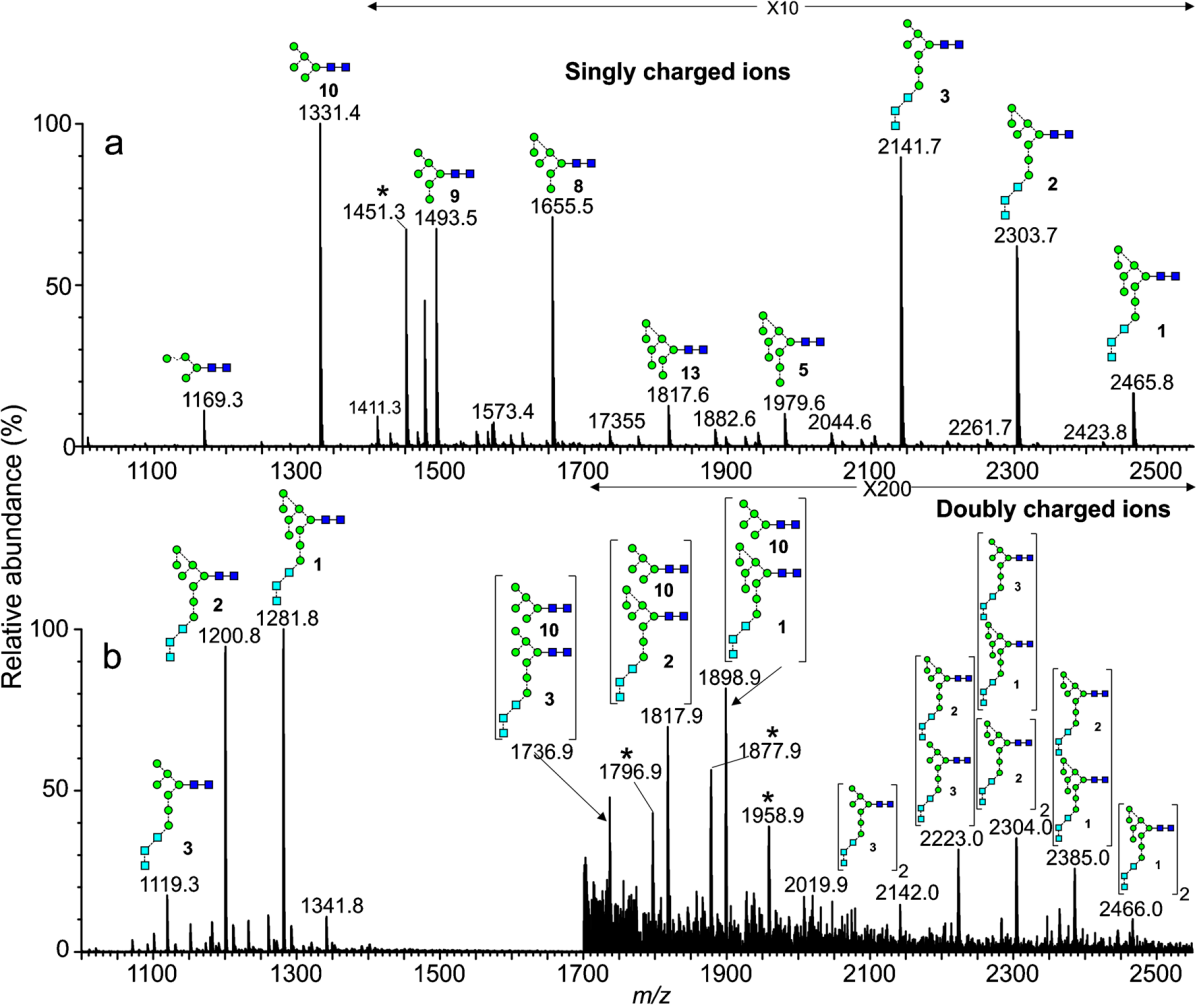
Negative ion nESI spectra of *N*-glycans released from HIV gp120 recombinantly expressed in the presence of NBDNJ. (a) Mobility-extracted singly charged [M + H_2_PO_4_]^-^ ions. (b) Mobility-extracted doubly charged ions. Ions at *m/z* 1119, 1200, and 1281 are [M + (H_2_PO_4_)_2_]^2-^ ions; the others are [M_2_ + (H_2_PO_4_)_2_]^2-^ ions. Peaks marked with an asterisk are [M + H_2_PO_4_ + HNaPO_4_]^2-^ ions (singly charged) and [M_2_ + H_2_PO_4_ + HNaPO_4_]^2-^ ions (doubly charged) (note magnification factors in the upper mass ranges)

Another example of where [M_2_ + (H_2_PO_4_)_2_]^2-^) ions were observed from viral glycans was in a sample of *N*-glycans released from HIV gp120 produced in the presence of *N*-butyldeoxynojirimycin (NBDNJ), a compound that blocks removal of the glucose residues from the Glc_3_Man_9_GlcNAc_2_ high-mannose glycan (**1**) originally attached to proteins during the biosynthesis of glycoproteins (Scheme 1). Consequent biotransformation by α-mannosidase removes the mannose residues from the d2 and d3 antennae to give Glc_3_Man_8_GlcNAc_2_ (**2**) and Glc_3_Man_7_GlcNAc_2_ (**3**, Scheme 1, Path A). The profile of released glycans from this sample is shown in Fig. 2. The doubly charged profile (Fig. 2b) shows, at the low mass end, the [M + (H_2_PO_4_)_2_]^2-^ ions, clearly showing preference for the higher mass glycans. The other ions are [M_2_ + (H_2_PO_4_)_2_)]^2-^ ions. The second of the two groups consist of dimers of the Glc_3_Man_7-9_GlcNAc_2_ glycans (**1** – **3**) with the first set being combinations of the Glc_3_Man_7-9_GlcNAc_2_ glycans with the very abundant Man_5_GlcNAc (**10**). Peaks marked with an asterisk are analogues of these ions with an additional HPO_4_Na, an adduct often seen in the spectra of phosphate adducts.

#### Ions of type [M – H_n_]^n-^ and [M + (H_2_PO_4_)_n_]^n-^ from other glycoproteins

**Fig. 3:**
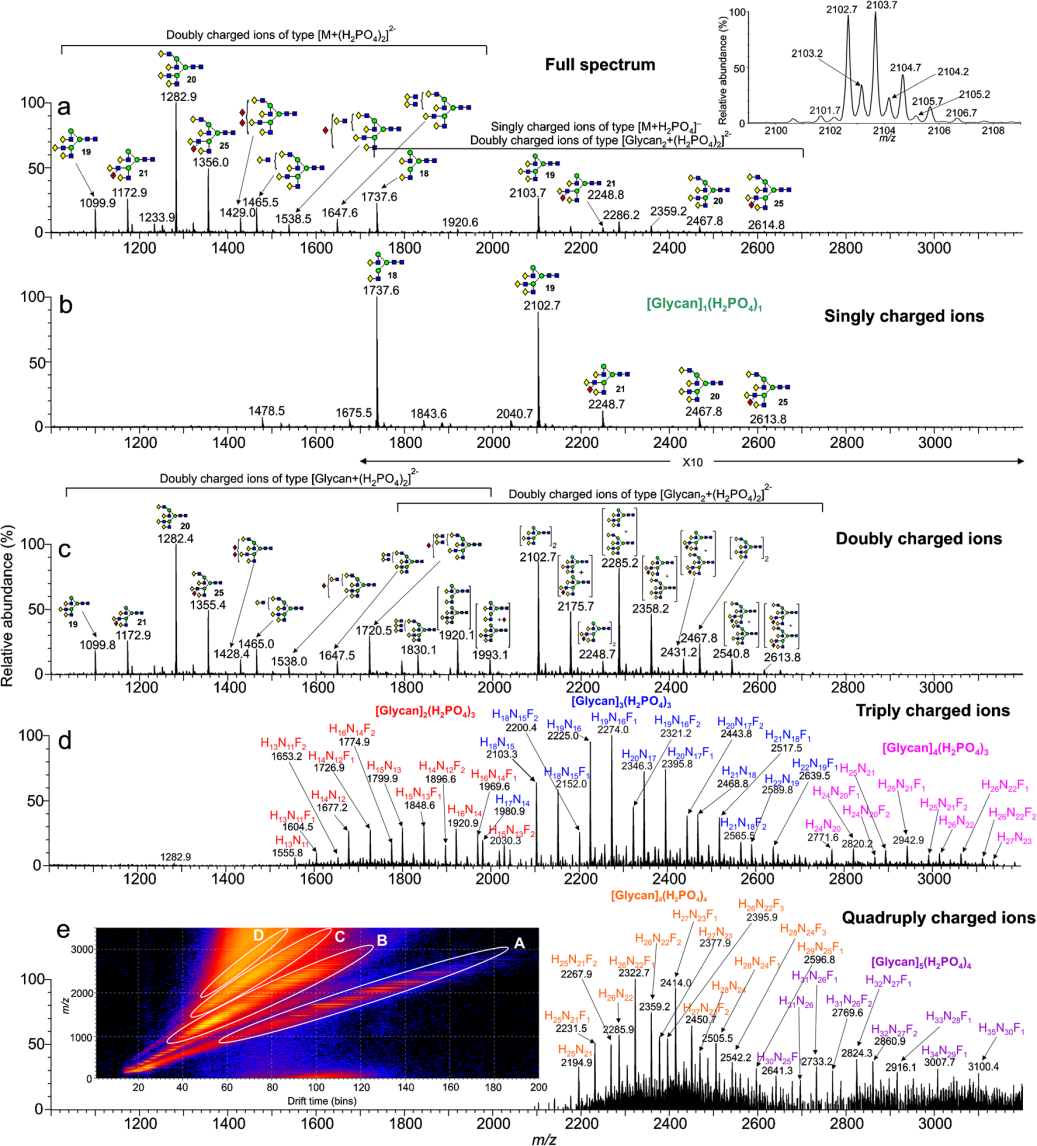
Negative ion nESI spectra of glycans released from AGP and desialylated. (a) Total profile. The inset shows an expansion of the ion at *m/z* 2102. (b) Mobility-extracted singly charged ions (M + H_2_PO_4_]^-^). (c) Mobility-extracted doubly charged ions ([M + (H_2_PO_4_)_2_]^2-^ and [M_2_ + (H_2_PO_4_)^2-^). (d) Mobility-extracted triply charged ions ([M_2_ + (H_2_PO_4_)_3_]^3-^, [M_3_ + (H_2_PO_4_)_3_]^3-^, [M_4_ + (H_2_PO_4_)_3_]^3-^). (e) Mobility-extracted quadruply charged ions ([M_4_ + (H_2_PO_4_)_4_]^4-^, [M_5_ + (H_2_PO_4_)_4_]^4-^). The inset shows the DriftScope profile; A = singly charged ions, B = doubly charged ions, C = triply charges ions, D = quadruply charged ions

The ability of ion mobility to extract multiply charge ions, often from a “noisy” region of the spectrum, provided a method for identification of large glycans, often present in low abundance and was investigated with several other glycan mixtures. Figure 3 shows the profile of the *N-*glycans released from desialylated AGP. Figure 3a shows the total profile and Fig. 3b and c show the mobility-extracted singly and doubly charged ions respectively. The [M + (H_2_PO_4_)_2_]^2-^ ions revealed the presence of several large glycans (*m/z* 1428.4, 1465.0, 1538.0, 1647.5, 1720.5, and 1830.1), containing extra fucosylation and several additional Gal-GlcNAc moieties that did not appear in the singly charged profile.

**Fig. 4:**
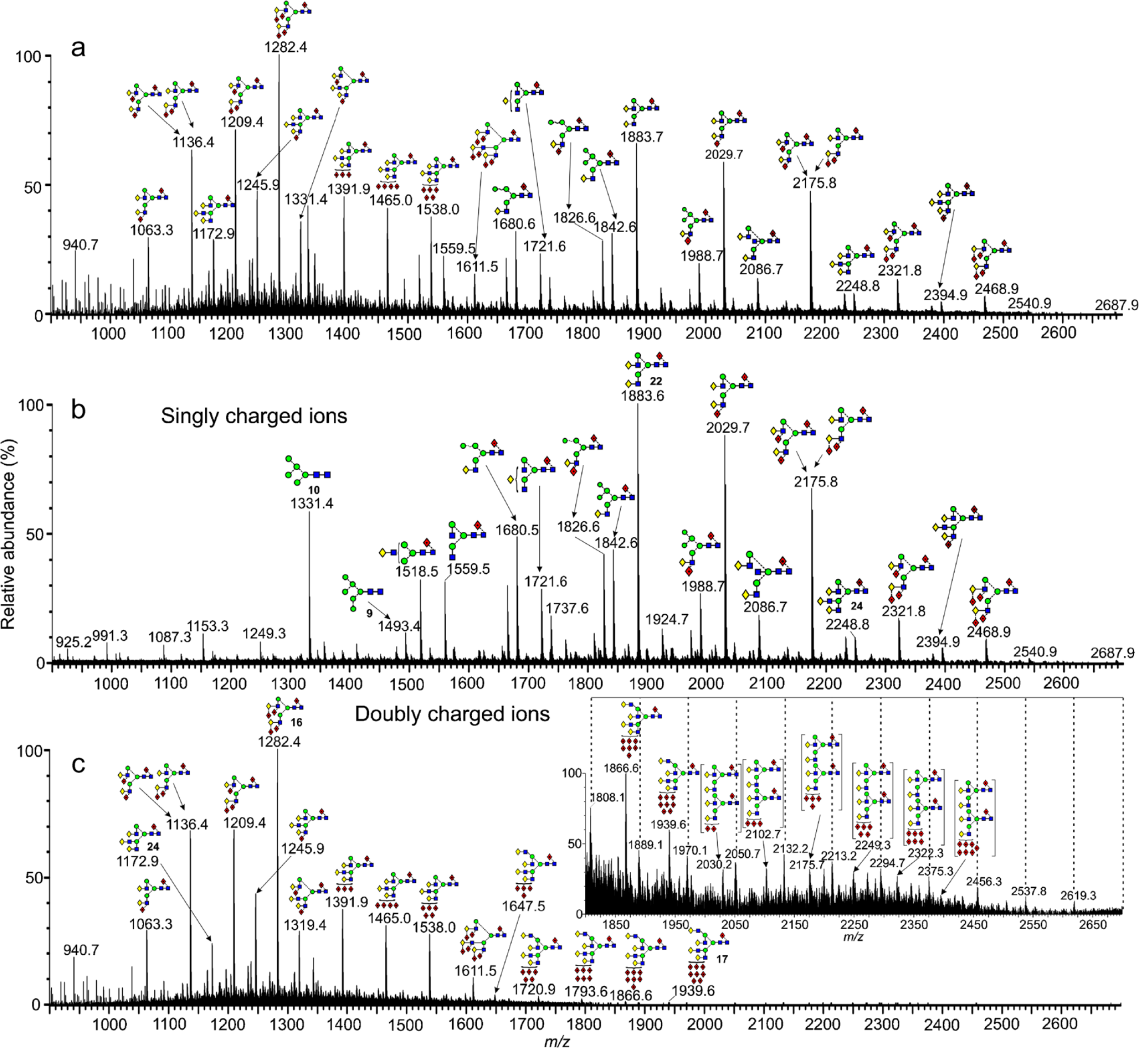
Negative ion nESI spectra of *N*-Glycans released from parotid gland glycoproteins. (a) Total profile. (b) Mobility-extracted singly charged ions (M + H_2_PO_4_]^-^). (c) Mobility-extracted doubly charged ions ([M + (H_2_PO_4_)_2_]^2-^). The inset shows a vertically magnified region from *m/z* 1800 showing doubly charged [M_2_ + (H_2_PO_4_)_2_]^2-^ ions and a series of unidentified ions separated by hexose units (dashed lines

A similar pattern of doubly charged ions was seen with the fucosylated glycans released from glycoproteins obtained from human parotid glands (Fig. 4). The profile of the singly charged glycans (Fig. 4b) was dominated by bi-antennary and hybrid glycans with varying numbers of fucose residues and containing relatively weak signals from the more highly fucosylated glycans. By contrast, the penta-fucosylated bi-antennary glycan (**16**) dominated the spectrum of the doubly charged ions (Fig. 4c) with additional sets of polyfucosylated tri- and tetra-antennary glycans, a few of which, such as the nona-fucosylated tetra-antennary glycan (**17**), do not appear to have been reported before from salivary glycoproteins [46, 58, 59]. The inset to panel c shows a vertically enlarged portion of the upper mass range. Also, marked with broken lines is a series of ions attributable to hexose oligomers but the specific compounds producing these ions were not identified.

#### Ions of type [M_n_ +(H_2_PO_4_)_n_]^n-^

Most reference glycans were observed to form [M_2_ + (H_2_PO_4_)]^2-^ ions when examined at concentrations greater than about 1 μg/μL. In addition, the larger ones, such as Glc_1_Man_9_GlcNAc_2_ (**4**), were found to form even larger clusters at higher charge states such as [M_3_ + (H_2_PO_4_)_3_]^3-^ and [M_4_ + (H_2_PO_4_)_4_]^4-^, that were easily separable by ion mobility. Glycans from AGP were also found to form doubly, triply, and quadruply charged ions as shown above in Fig. 3c, d, and e. The dimeric ion formed from two triantennary glycans (**19**, *m/z* 2102) from AGP was detected by its isotope peaks that were clearly visible among the peaks from the singly charged ion (inset, Figs. 2a and 3a). Mixed dimeric ions appeared at *m/z* values such as *m/z* 1920 ((Hex_5_GlcNAc_4_) + Hex_6_GlcNAc_5_) + (H_2_PO_4_)_2_) and at *m/z* 2285.3 ((Hex_6_GlcNAc_5_) + (Hex_7_GlcNAc_6_) + (H_2_PO_4_)_2_). The ion in Fig. 3c at *m/z* 2175.7 contains one fucose residue additional to the dimer at *m/z* 2102.4 and was formed by a combination of the triantennary glycan **19** and its fucosylated analog (mainly **21**). The ion at *m/z* 2248.7 contains two fucose residues and a composition corresponding to two triantennary glycans. Compositions of the higher mass ions are as labelled in the figure. Combinations with the larger glycans greatly increased the relative abundance of these ions making the presence of these larger glycans easier to detect than in the spectra of the singly charged glycans. Three types of triply charged ions were observed (Fig. 3d) with compositions of [M_2_ + (H_2_PO_4_)_3_]^3-^, [M_3_ + (H_2_PO_4_)_3_]^3-^, and [M_4_ + (H_2_PO_4_)_3_]^3-^, and quadruply charged ions appeared as [M_4_ + (H_2_PO_4_)_4_]^4-^, [M_5_ + (H_2_PO_4_)_4_]^4-^ species. Further investigations of all of these ions were made by CID as discussed below.

### CID spectra

#### Singly charged ions

**Fig. 5:**
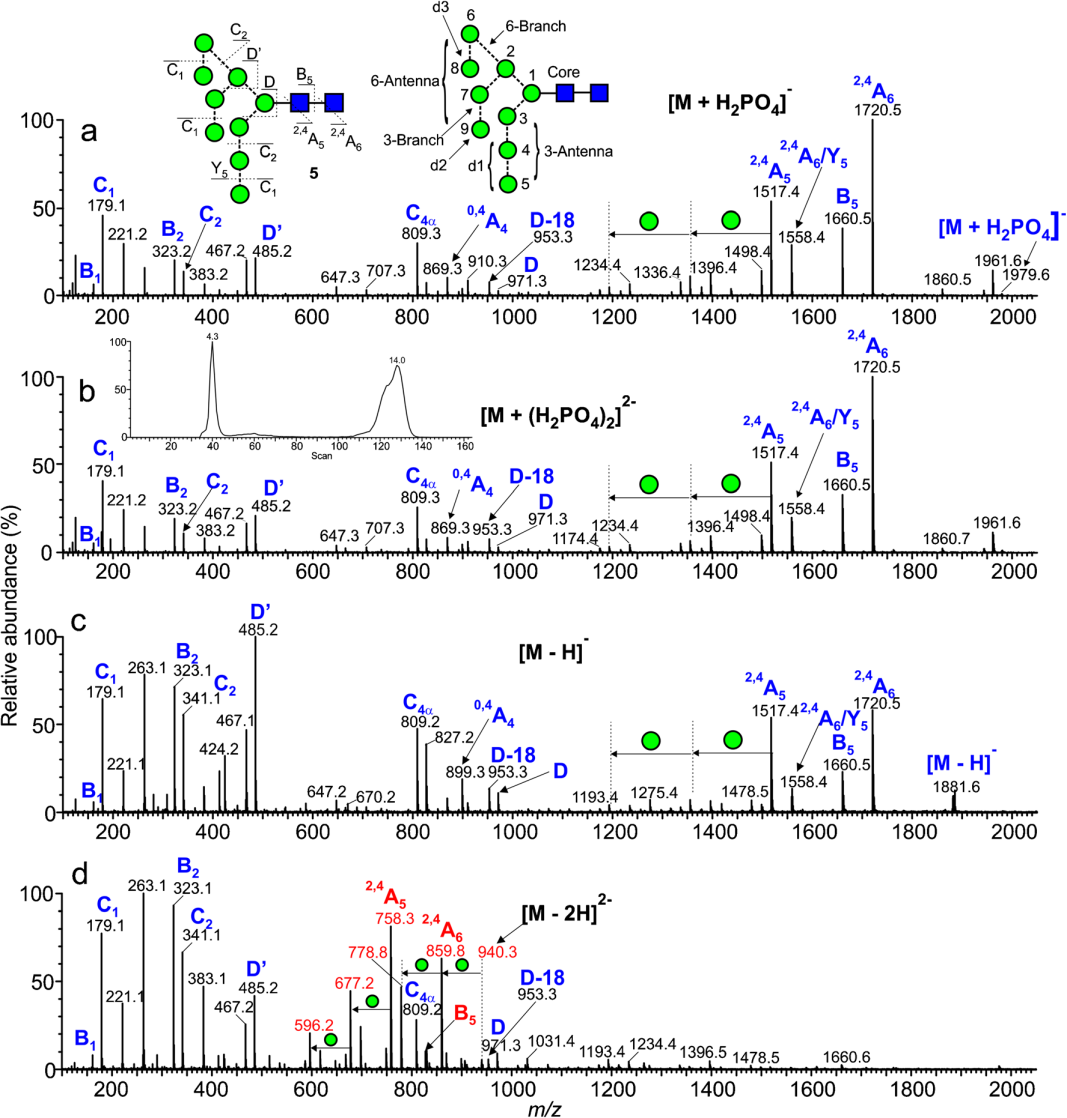
Negative ion CID spectra of the high-mannose glycan Man_9_GlcNAc_2_ (**5**). (a) [M + H_2_PO_4_]^-^ ion. (b) M + (H_2_PO_4_)_2_]^2^^-^ ion. (c) [M – H]^-^ ion. (d) [M – H_2_]^2-^ ion. Doubly charged ions are shown in red. The inset to panel b shows the ATD profile with drift times as ms

**Fig. 6:**
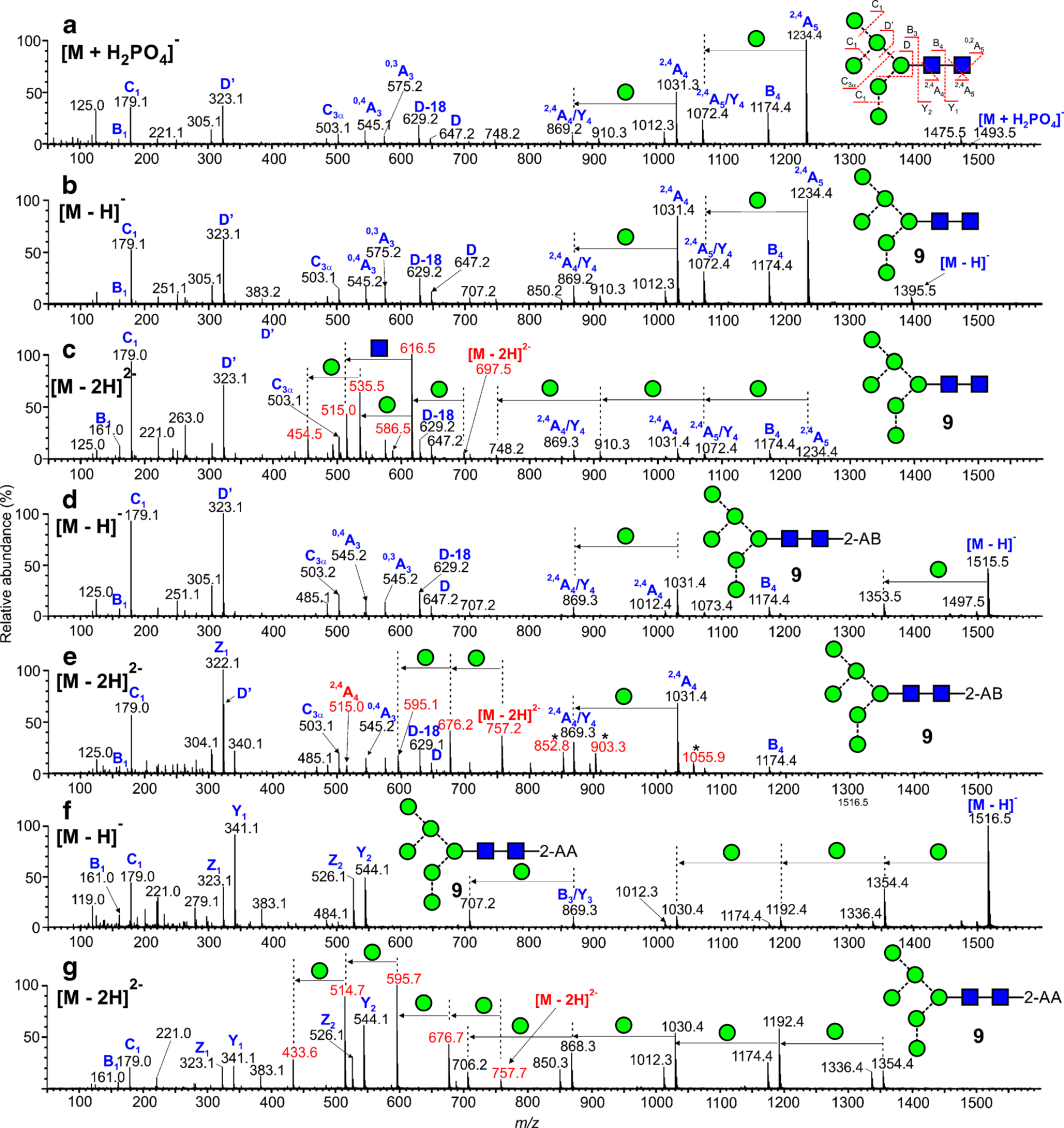
Negative ion CID spectra of the high-mannose glycan Man_6_GlcNAc_2_ (**9**). (a) [M + H_2_PO_4_]^-^, (b) [M – H]^-^, (c) [M – H_2_]^2-^, (d) [M – H]^-^ of 2-AB derivative, (e) [M – H_2_]^2-^ of 2-AB derivative (peaks marked with an asterisk appear to be contaminants), (f) [M – H]^-^ of 2-AA derivative, (g) [M – H_2_]^2-^ of 2-AA derivative. Doubly charged ions are shown in red

The CID spectra of the singly charged [M – H]^-^ ions of the neutral glycans paralleled that of the [M + H_2_PO_4_]^-^ ions because the first stage of fragmentation of the latter ions involves proton removal by the anion to leave the [M - H]^-^ ion. Representative spectra are shown in Fig. 5a (M_9_GlcNAc_2_, [M + H_2_PO_4_]^-^ ion), Fig. 5c (Man_9_GlcNAc_2_, [M – H]^-^ ion), Fig. 6a (Man_6_GlcNAc_2_, [M + H_2_PO_4_]^-^ ion), and Fig. 6b (Man_6_GlcNAc_2_, [M – H]^-^ ion). The nature of the Cl^-^, NO_3_^-^, and H_2_PO_4_^-^ anions was found to make negligible difference on the appearance of the spectra although larger anions such as Br^-^ and I^-^ produced less fragmentation. Full details of the diagnostic ions that can be formed in these negative ion spectra can be found in earlier publications [20, 52–55, 60]. Briefly, referring to Fig. 5a, the structure of the *N*,*N’*-di-acetylchitobiose core region, including the presence of absence of fucose, was defined by ^2,4^A_R_, B_R-1_, and ^2,4^A_R-1_ ions (*m/z* 1720.5, 1660.5, and 1517.4, respectively); the non-reducing residues produced C_1_ fragments (*m/z* 179) and the composition of the 6-antenna was defined by D and D-18-type ions (*m/z* 971.3 and 953.3 respectively), formed by loss of the *N*,*N’*-di-acetylchitobiose core and the d1 antenna (also called the 3-antenna), accompanied by ^0,4^A_3_ and ^0,3^A_3_ cross-ring fragments at *m/z* 899.3 and 869.3, respectively, appearing in the center region of the spectra (terms such as 3-antenna and d1 antenna are defined in Fig. 5a).

#### Doubly charged ions of type [M + (H_2_PO_4_)_2_]^2-^

Doubly charged [M + (H_2_PO_4_)_2_]^2-^ ions fragmented to give almost entirely singly charged products such that the spectra were almost indistinguishable from those of the singly charged [M + H_2_PO_4_]^-^ ions as illustrated with the high-mannose glycans Man_9_GlcNAc_2_ (**5**, Fig. 5a, b). A few very minor (< 2% RI) doubly charged fragments such as the ^2,4^A_R_ ion were sometimes seen at lower cone voltages. The mechanism would appear to be loss of H_2_PO_4_^-^ leaving the [M + H_2_PO_4_]^-^ ion which fragmented as the singly charged ion. Thus, structural interpretations of the larger glycans are often more appropriately made with the doubly charged [M + H_2_PO_4_]^2-^ ions when their relative abundance exceeds that of the corresponding singly charged ones. These [M + (H_2_PO_4_)_2_]^2-^ ions were not totally stable using the Waters G2Si instrument. Although the doubly charged ion was selected in the quadrupole, ion mobility, after the trap cell, showed both this ion and the singly charged [M + H_2_PO_4_]^-^ species as shown by the arrival time distribution (ATD) profile (inset to Fig. 5b). The CID spectrum of this [M + H_2_PO_4_]^-^ ion was identical to that of the singly charged [M + H_2_PO_4_]^-^ ion from a reference sample. The asymmetry of the ATD peak appeared to be caused by anomers because all fragment ions exhibited the same profile [61] and reducing end reduction produced a symmetrical profile [62].

#### Doubly charged ions of type [M –H_2_]^2-^

The CID spectra of these ions differed considerably from those of the and [M – H]^-^ ions as can be seen from Figs. 5c and d and 6b and c. Doubly charged ions dominated the spectra. In particular, the ^2,4^A_R_, B_R-1_, and ^2,4^A_R-1_ ions appeared mainly as doubly rather than singly charged ions (*m/z* 859.8, 829.8, and 758.3 in Fig. 5d and at *m/z* 616.7, 586.7, and 515.2, respectively, in Fig. 6c). In the spectra of the high-mannose glycans, successive losses of mannose residues from the ^2,4^A_R_ ion produced major singly charged fragments but no such loss was observed from the molecular ion. In contrast to this observation, the spectrum of reduced Man_6_GlcNAc_2_ reported by Tjondro et al. [63], where formation of the ^2,4^A_R_ ion was blocked by the open ring, showed a very prominent loss of mannose directly from the doubly charged molecular ion. The other main diagnostic ions tended to be singly charged. Thus, in the CID spectra of the [M – H_2_]^2-^ ion from Man_6_GlcNAc_2_ (**9**, Fig. 6c), the D, D-18, ^0,3^A_3_, and ^0,4^A_3_ ions appeared at *m/z* 647.2, 629.2, 575.2, and 545.2 respectively albeit of lower relative abundance than in the spectrum of the doubly charged phosphate adduct. C_1_ and C_2_ ions were also singly charged (*m/z* 179.1 and 341.1 respectively).

**Fig. 7:**
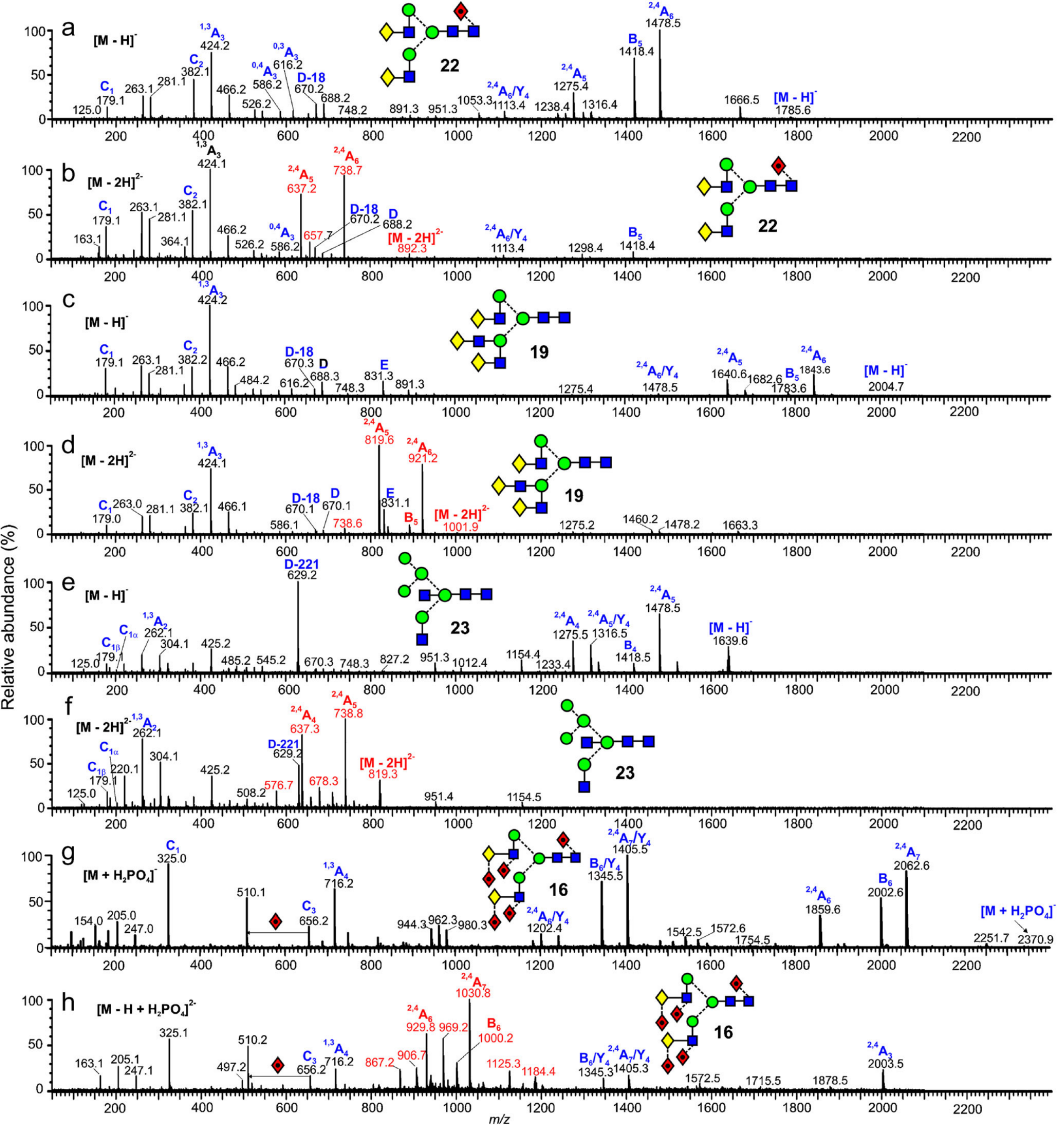
Comparison of the singly ([M – H]^-^, panels a, c, e, and g) and doubly ([M – H_2_]^2-^, panels b, d, f, and h) ions from four complex glycans. Panel g shows the spectrum of the [M – H + (H_2_PO_4_)]^2-^ ion in the absence of the [M – H]^2-^ ion

Figure 7 shows a selection of CID spectra of the [M – H_2_]^2-^ ions from four other types of *N*-glycans and contrasts three of them with their [M – H]^-^ counterparts. The [M – H]^-^ spectrum of the penta-fucosylated bi-antennary glycan (**16**) was not available, so the [M + H_2_PO_4_]^-^ spectrum is shown (Fig. 7g). Diagnostic ions from the biantennary glycan (**22**, Fig. 7a) were the C_1_ fragment at *m/z* 179 (non-reducing terminal galactose), the ^1,3^A_3_ ion at *m/z* 424 ([Gal-GlcNAc-O-CH=CH_2_-OH]^-^), the D, D-18, ^0,3^A_3_, and ^0,4^A_3_ ions at *m/z* 688.2, 670.2, 616.2, and 586.2, respectively, and the ^2,4^A_6_, B_5_, and ^2,4^A_5_ ions at *m/z* 1478.5, 1418.4, and 1275.4, respectively. The masses of the latter three ions, which all lack fucose, confirm the presence of fucose at the 6-position of the reducing-terminal GlcNAc residue. These ions are all present in the spectrum of the [M – H_2_]^2-^ ion (Fig. 7b), although the ^2,4^A_6_, B_5_, and ^2,4^A_5_ ions appear as doubly charged fragments as they did in the spectra of the high-mannose glycans above. This pattern of fragmentation is similar to the spectrum reported by Ni et al. [64] using an Agilent 6520 Q-TOF instrument although the relative abundance of some of the ion differed.

Diagnostic ions in the spectra of the triantennary glycan (**19**, Fig. 7c and d) (particularly *m/z* 831, labelled E [60]) and in that of the bisected glycan (**23**, Fig. 7e and f, specifically the D-221 fragment at *m/z* 629) are present in the spectra of both the [M – H]^-^ and [M – H_2_]^2-^ ions, all as singly charged fragments. Also, in common with the spectra of other [M – H_2_]^2-^ ions, the ^2,4^A_6_, B_5_, and ^2,4^A_5_ ions appeared as doubly charged fragments in the spectra of the [M – H_2_]^2-^ ions. Figure 7h shows the CID spectrum of the [M – H + (H_2_PO_4_)]^2-^ ion (the spectrum of the [M – H_2_]^2-^ ion was not available) from a penta-fucosylated biantennary glycan (**16**). Loss of the phosphate gave [M – H]^-^ which produced all of the necessary diagnostic fragments for its identification. Highlighted ions are C_2_ (*m/z* 325, Fuc-Gal) and the ^1,3^A_4_ cross-ring fragment at *m/z* 716 ([Gal-GlcNAc-O-CH=CH_2_-OH]^-^ with two fucose residues). Thus, these results show that the spectra of [M – H_2_]^2-^ ions, along with those of the [M – H]^-^ and [M + H_2_PO_4_]^-^ ions, contain all of the diagnostic fragments reported earlier that are necessary for characterization of these glycans.

#### 2-Aminobenzamide (2-AB) derivatives

2-AB derivatives, prepared by reductive amination, are commonly used as fluorescent analogues of these glycans for detection in HPLC experiments [65]. They are often encountered in MS work even though the fluorescent tag is not needed. Figure 6d shows the CID spectrum of singly charged phosphate adduct of Man_6_GlcNAc_2_ (**9**) derivatized in this manner. The spectrum of the lower mass region of the spectrum mirrors that of the underivatized glycan (Fig. 6a) but, because of the open-ring nature of the reducing terminal GlcNAc residue (a consequence of the reductive amination reaction during labelling), the ^2,4^A_4_ ion, which is important in identifying the presence of fucose attached to this residue (see below), was missing. The CID spectrum of the [M – H_2_]^2-^ ion (Fig. 6e) contained the same diagnostic singly charged ions in the low mass region and, like the spectrum of the reduced glycan [63], successive losses of mannose residues (*m/z* 676.2, 595.1, 514.1) were prominent from the molecular ion. In the spectra of the [M – H_2_]^2-^ ions from two isomers of Man_7_GlcNAc_2_ as 2-aminopyridine (2-AP) derivatives reported by Yan et al. [21], the most abundant fragment was loss of mannose from the molecular ion (doubly charged). However, the spectrum of the d1,d3-isomer appeared to lack the D and D-18 fragment ions. In the spectra of these two isomers, and in that of the 2-AB derivative of Man_6_GlcNAc_2_ (**9**), the ^2,4^A_R-1_ (^2,4^A_4_ at *m/z* 1031.4 in Fig. 6e) ion was observed mainly as a singly charged fragment. The most abundant ion was the Y_1_ fragment (GlcNAc-2-AB) at *m/z* 322.1.

#### 2-Aminobenzoic acid (2-AA) derivatives

2-AA derivatives [66] have received increasing popularity in recent years [67] because of their ease of preparation and high fluorescence. The incorporation of an acidic group potentiates formation of deprotonated molecules and localizes the negative charge. Although providing high negative ion sensitivity, localization of the charge on the derivative considerably alters the fragmentation from that discussed above. Thus, the singly charged spectrum of Man_6_GlcNAc_2_ (**9**, Fig. 6f) contained mainly Y-type fragments produced by losses from the non-reducing terminus. There was very little information on the topology of the antennae except a moderate increase in the relative abundance of the Y_3α_ ion (loss of the d2 and d3 antennae) from the larger high-mannose glycans. The diagnostic ^2,4^A ions from the reducing terminus and the D-type ions were missing. Fragmentation of the [M – H_2_]^2-^ ions from the high-mannose ions was even less informative (Fig. 6g). Although prominent singly charged ions were produced from successive losses of mannose residues, there was no information available regarding the glycan topology. For more information on the fragmentation of 2-AA-derivatized glycans, see Harvey [68].

#### Multiply charged ions of type [M_n_ + (H_2_PO_4_)_n_]^n-^ from single glycans

**Fig. 8:**
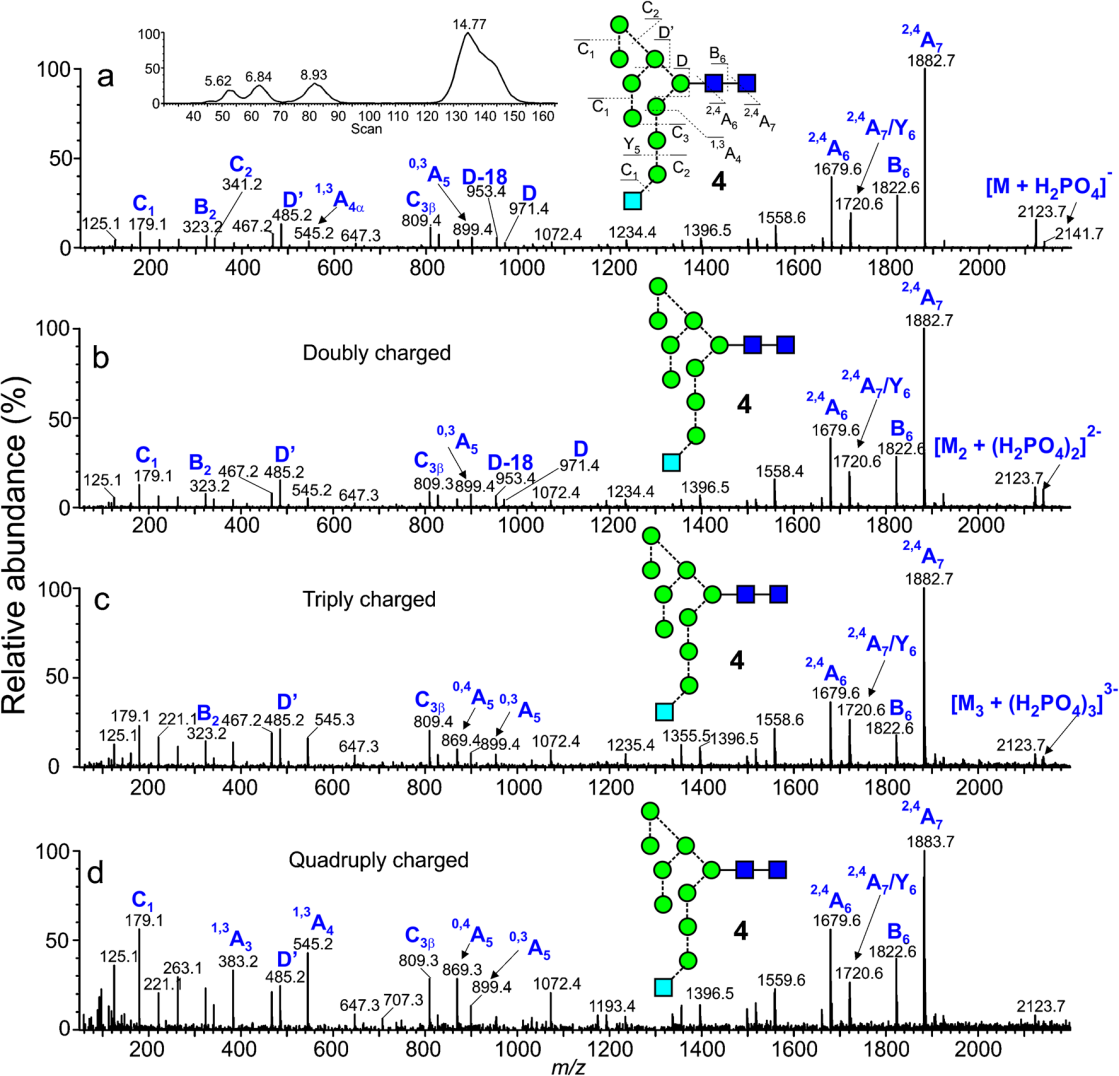
Negative ion CID spectrum of (a) the singly charged [M + H_2_PO_4_]^-^ ion, (b) the doubly charged [M_2_ + (H_2_PO_4_)_2_]^2-^ ion, (c) the triply charged [M_3_ + (H_2_PO_4_)_3_]^3-^ ion, and (d) the quadruply charged [M_4_ + (H_2_PO_4_)_4_]^4-^ ion from Glc_1_Man_5_GlcNAc_2_ from Glc_1_Man_5_GlcNAc_2_ (**4**). The inset to panel a shows the ATD profile of the ion at *m/z* 2141.7. Numbers on the ATD peaks are ms

The spectra of several reference glycans were examined. These included biantennary glycans with zero (**26**), one (**27**), and two (**18**) galactose residues, with (**28-30**) and without (**26**, **27**, **18**) a bisecting GlcNAc residue and with or without core fucose, a hybrid glycan (**23**), and a series of high-mannose glycans. The relative amount of the dimeric ions in the total glycan profile tended to increase with increasing molecular weight but the fragmentation spectra of all dimers were similar to those of the monomers. Glc_1_Man_9_GlcNAc_2_ (**4**) formed dimers, trimers, and tetramers that were well-separated by ion mobility as shown by the ATD profile (inset Fig. 8a). CID spectra of the dimer and monomer were identical but the trimer and tetramer showed progressively increasing relative abundance of the ions in the low mass region, particularly ^1,3^A fragment ions from the 3-antenna (*m/z* 545.2 and 383.2) as shown in Fig. 8, reflecting greater instability.

#### Samples with multiple glycans

**Fig. 9:**
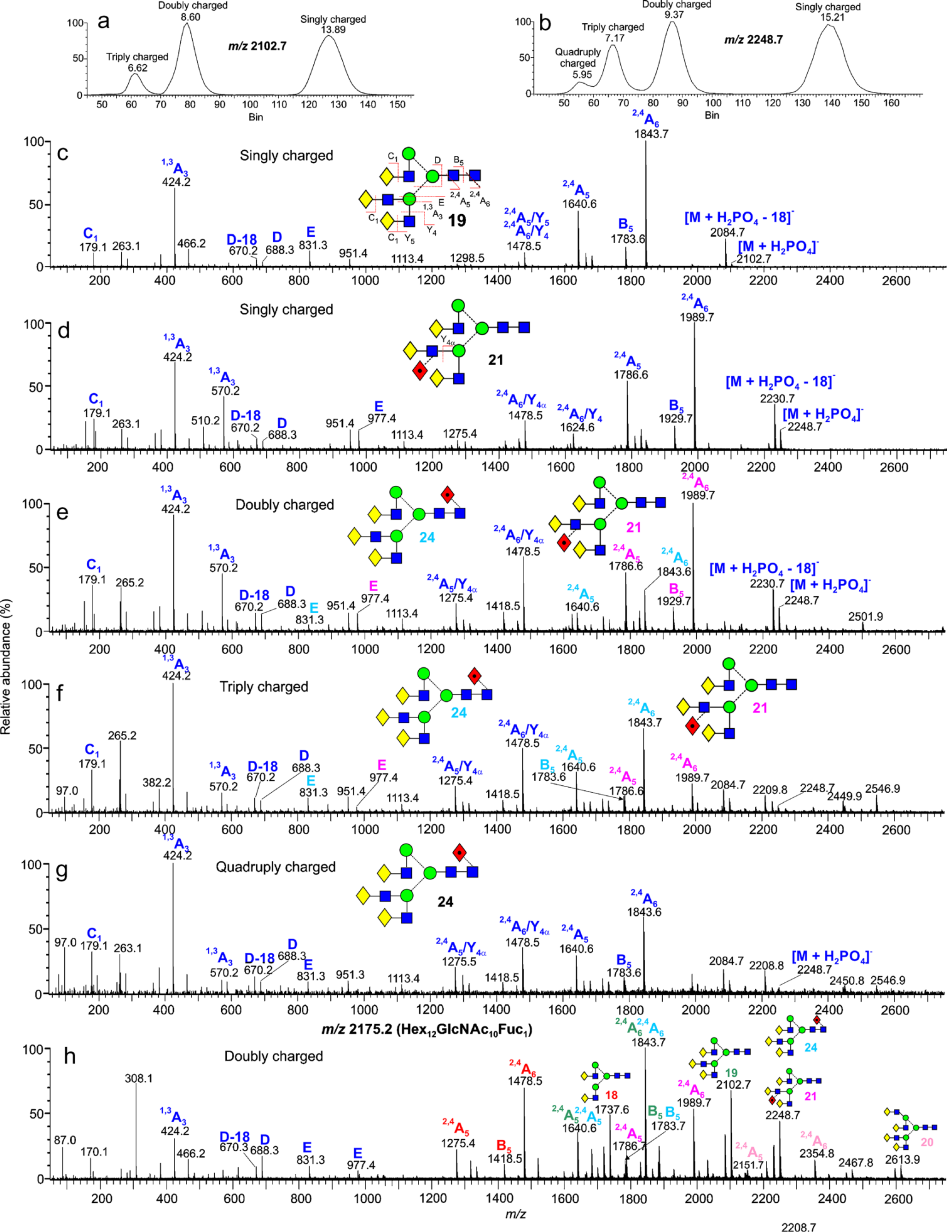
(a) ATD profile of ions in the CID spectrum of *m/z* 2102.7 from AGP (M_n_ + (H_2_PO_4_)_n_)^n-^ ions). (b) ATD profile of ions in the CID spectrum of *m/z* 2248.7 from AGP ([M_n_ + (H_2_PO_4_)_n_]^n-^ ions). (c) The negative ion CID spectrum of the triantennary glycan **19**. (d–g) Negative ion CID spectra of (d) the singly charged ([M + (H_2_PO_4_)]^-^) ions, (e) the doubly charged ([M_2_ + (H_2_PO_4_)_2_]^2-^) ions, (f) the triply charged ([M_3_ + (H_2_PO_4_)_3_]^3-^) ions, and (g) the quadruply charged ([M_4_ + (H_2_PO_4_)_4_]^4-^) ions at *m/z* 2175.7 from AGP. Fragment ions indicating the location of the fucose residue on the 3-antenna are shown in color for each glycan. (h) Negative ion CID spectrum of the doubly charged ion ([Hex_12_GlcNAc_10_Fuc_1_ + (H_2_PO_4_)_2_]^2-^) at *m/z* 2176.2 from AGP. Several constituents contribute to the ion

Samples containing several types of glycan, such as those released from AGP, additionally produce multimeric charged ions with mixtures of glycans. The most abundant glycan (following desialylation) released from AGP is the triantennary complex glycan (**19**, Hex_6_GlcNAc_5_). The molecular ion ([M + H_2_PO_4_]^-^ (*m/z* 2102.7) in the spectrum of our sample clearly showed the presence of [M_2_ + (H_2_PO_4_]_2_]^2-^ ions, and ion mobility separated three sets of ions (Fig. 9a) with charge states 1, 2, and 3. Their negative ion CID spectra were virtually identical (Fig. 9c) except that the fragments formed by losses of Gal-GlcNAc (Y_4_ cleavage) from the ^2,4^A_6_ and ^2,4^A_5_ ion (*m/z* 1478.5 and 1275.4 respectively) were considerably more abundant in the spectra of the doubly and triply charged ions. The CID spectra were characterized by ^2,4^A_6_, B_5_, and ^2,4^A_5_ ions defining the core region, *m/z* 424 (^1,3^A_3_) containing Gal-GlcNAc from the antennae and *m/z* 831 (labelled ion E) which is the ion diagnostic of triantennary glycans branched on the 3-antenna as described above. Smaller glycans, such as the biantennary glycan (**18**), did not form dimers.

The negative ion CID spectrum of the fucosylated triantennary glycan from AGP (*m/z* 2248.7, singly charged) is shown in Fig. 9d. Location of the fucose residue on the 3-antenna is shown by the masses of the ^2,4^A_6_, B_5_, and ^2,4^A_5_ ions together with *m/z* 570 (^1,3^A_3_, [Gal-(Fuc)GlcNAc-O-CH=CH_2_-OH]^-^) and *m/z* 977 (E-ion + fucose). The ATD profile of *m/z* 2248.7 (Fig. 9b) showed three additional constitutes with charge states 2, 3, and 4. CID spectra are shown in Fig. 9e, f, and g. These spectra show an increasing contribution of the core fucosylated isomer (**24**) such that the quadruply charged ion (Fig. 9g, [M_4_ + (H_2_PO_4_)_4_]^4-^) consisted almost entirely of this isomer. Fragment ions are color coded to match the glycan number. Ions in these higher charge states, thus, possibly provide a means to confirm the presence of minor isomers in mixtures.

The situation becomes rather complicated when ions contain two or more constituents as shown in Fig. 9h, which shows the CID spectrum of the doubly charged ion at *m/z* 2175.2 from AGP with a composition of Hex_12_GlcNAc_10_Fuc_1_. Molecular ions in the spectra at *m/z* 1737.6, 1883.7, 2102.7, 2248.7, and 2613.9 show the presence of glycans **18**, **22**, **19**, **21**, and **20**, respectively. ^2,4^A_6_, B_5_, and ^2,4^A_5_ ions for each glycan are shown in color. Thus, the ion consists of a mixture of the glycans **20**/**22**, **19**/**24**, and **18** with probably a fucosylated tetraantennary glycan such as **25**, although its molecular ion was absent from the spectrum.

Figure 3d shows the even more complex profile from the triply charged ions from trimers where there are more opportunities for the formation of mixed trimers. Constituents are labelled in the figure. The relative abundance of trimers containing fucose was much greater than in the spectra of the singly charged ions making their presence easier to detect.

## Conclusions

Under negative ESI conditions, most *N*-glycans form singly charged ions of the type [M – H]^-^ or [M + adduct]^-^ where the adduct can be anions such as halogens, phosphate, or nitrate. Higher mass glycans tend to form doubly charged ions. In many samples, in very large glycans, such as tetra-antennary compounds bearing *N*-acetyl-lactosamine extensions, only doubly charged ions were seen. Although not reported in this paper, it has been found that very large high-mannose *N*-glycans found in some fungi produce triply and quadruply charged ions and that no singly charged ions from these larger glycans are present in the spectra. In addition to these ions, small quantities of dimeric doubly charged ions of structure [M_2_ + (H_2_PO_4_)_2_]^2-^ can also be produced and, in some cases, tri- and tetrameric ions in the appropriate charge states.

CID spectra of the [M – H]^-^ and [M + adduct]^-^ ions, where the adduct is phosphate, chloride, or nitrate, tend to be almost identical because the first stage of fragmentation by the adducted glycans is removal of a proton (loss of HA, where A is the adduct) to leave the [M – H]^-^ ion, which is then further fragmented. Fragmentation of the doubly charged [M + (H_2_PO_4_)_2_]^2-^ ion is similar but the [M – H_2_]^2-^ ions produce large amounts of doubly charged fragment ions (^2,4^A_R_, B_R-1_, and ^2,4^A_R-1_) from the reducing end of the molecule. Most other structurally diagnostic ions, such as the D and D-18 ions, remain singly charged and appear in the spectra of the doubly charged precursors.

In addition to these ions, small quantities of dimeric doubly charged ions of structure [M_2_ + (H_2_PO_4_)_2_]^2-^ can also be produced from the phosphate adducts. M can be two molecules of a single glycan or one each of two different molecules. If the starting concentration of the glycan is sufficiently high, larger oligomers of the type [M_3_ + (H_2_PO_4_]_2_]^3-^ and [M_4_ + (H_2_PO_4_)_4_]^4-^ can also form. Although the CID spectra of the singly and doubly charged ions are virtually identical, those of the larger charge states show an increase in the relative abundance of the lower mass fragments consistent with their being more sensitive to the collision energy, although, in mixed dimers, it appeared that there is a fairly random mixture of the two constituents.

In conclusion, ion mobility enables multiply charged ions to be extracted from the spectra of glycan mixtures, often enabling new compounds to be identified, particularly when they are present at low concentration. CID spectra contain the same diagnostic ions as found in the spectra of the singly charged ions, thus providing a useful addition to the ion mobility/negative ion technique reported earlier.

## Data Availability

Not applicable

## Abbreviations

2-AA: 2-Aminobenzoic acid
2-AB: 2-Aminobenzamide
2-AP: 2-Aminopyridine
AGP: α1-Acid glycoprotein
ATD: Arrival time distribution
CHO: Chinese hamster ovary
CID: Collision-induced dissociation
FAB: Fast-atom bombardment
Fuc: Fucose
Gal: Galactose
GalNAc: *N*-Acetylgalactosamine
GC/MS: Gas chromatography/mass spectrometry
Glc: Glucose
GlcNAc: *N*-Acetylglucosamine
HEK: Human embryonic kidney
Hex: Hexose
HIV: Human immunodeficiency virus
HPLC: High-performance liquid chromatography
MALDI: Matrix-assisted laser desorption/ionization
Man: Mannose
MS: Mass spectrometry
NBDNJ: *N*-Butyldeoxynojirimycin
nESI: Nanoelectrospray ionization
Neu5Ac: *N*-Acetylneuraminic acid
PNGase: Peptide *N*-glycosidase
Q: Quadrupole
RF: Radiofrequency
SARS: Severe acute respiratory syndrome
TOF: Time-of-flight
TWIMS: Travelling wave ion mobility mass spectrometry

## References

[1] Varki A (2017). Biological roles of glycans. Glycobiology.

[2] Harvey DJ, Merry AH, Royle L, Campbell MP, Dwek RA, Rudd PM (2009). Proposal for a standard system for drawing structural diagrams of *N*- and *O*-linked carbohydrates and related compounds. Proteomics.

[3] Harvey DJ (2001). Identification of protein-bound carbohydrates by mass spectrometry. Proteomics.

[4] Harvey DJ, Cole RB (2010). Carbohydrate analysis by ESI and MALDI. Electrospray and MALDI mass spectrometry: fundamentals, instrumentation, practicalities, and biological applications.

[5] Alley WR, Mann BF, Novotny MV (2013). High-sensitivity analytical approaches for the structural characterization of glycoproteins. Chem Rev.

[6] Harvey DJ, Roberts GCK (2013). Mass spectrometry of *N*-linked carbohydrates and glycoproteins. Encyclopedia of biophysics.

[7] Novotny MV, Alley WR, Mann BF (2013). Analytical glycobiology at high sensitivity: current approaches and directions. Glycoconj J.

[8] Harvey DJ, Griffiths JR, Unwin RD (2017). Analysis of protein glycosylation by mass spectrometry. Analysis of protein post-translational modifications by mass spectrometry.

[9] Gray C, Flitsch SL, Witczak ZJ, Bielski R (2017). Methods for the high resolution analysis of glycoconjugates. Coupling and decoupling of diverse molecular units in glycosciences.

[10] Ruhaak LR, Xu G, Li Q, Goonatilleke E, Lebrilla CB (2018). Mass spectrometry approaches to glycomic and glycoproteomic analyses. Chem Rev.

[11] Bagdonaite I, Vakhrushev SY, Joshi HJ, Wandall HH (2019). Viral glycoproteomes: technologies for characterization and outlook for vaccine design. FEBS Letts.

[12] Cipollo JF, Parsons LM (2020). Glycomics and glycoproteomics of viruses: mass spectrometry applications and insights toward structure-function relationships. Mass Spectrom Rev.

[13] Zhu H, Aloor A, Ma C, Kondengaden SM, Wang PG (2020). Mass spectrometric analysis of protein glycosylation. ACS Symp Ser.

[14] Dell A, Carman NH, Tiller PR, Thomas-Oates JE (1988). Fast atom bombardment mass spectrometric strategies for characterising carbohydrate-containing biopolymers. Biomed Environ Mass Spectrom.

[15] Harvey DJ (2021). Analysis of carbohydrates and glycoconjugates by matrix-assisted laser desorption/ionization mass spectrometry: an update for 2015-2016. Mass Spectrom Rev.

[16] Powell AK, Harvey DJ (1996). Stabilisation of sialic acids in *N*-linked oligosaccharides and gangliosides for analysis by positive ion matrix-assisted laser desorption-ionization mass spectrometry. Rapid Commun Mass Spectrom.

[17] Wheeler SF, Domann P, Harvey DJ (2009). Derivatization of sialic acids for stabilization in matrix-assisted laser desorption/ionization mass spectrometry and concomitant differentiation of α(2-3) and α(2-6) isomers. Rapid Commun Mass Spectrom.

[18] Vreeker GCM, Nicolardi S, Bladergroen MR, van der Plas CJ, Mesker WE, Tollenaar RAEM, et al. Automated plasma glycomics with linkage-specific sialic acid esterification and ultrahigh resolution MS. Anal Chem. 2018;90:11955–61. 10.1021/acs.analchem.8b02391 PMC6209171 30230816

[19] de Haan N, Yang S, Cipollo J, Wuhrer M. Glycomics studies using sialic acid derivatization and mass spectrometry. Natl Rev. 2020;4:229–42. 10.1038/s41570-020-0174-3 37127981

[20] Harvey DJ (2020). Negative ion mass spectrometry for the analysis of *N*-linked glycans. Mass Spectrom Rev.

[21] Yan S, Wang H, Schachter H, Jin C, Wilson IBH, Paschinger K (1862). Ablation of *N*-acetylglucosaminyltransferases in *Caenorhabditis* induces expression of unusual intersected and bisected *N*-glycans. Biochim Biophys Acta, Gen Subj.

[22] Ashwood C, Lin C-H, Thaysen-Andersen M, Packer NH (2018). Discrimination of isomers of released *N*- and *O*-glycans using diagnostic product ions in negative ion PGC-LC-ESI-MS/MS. J Am Soc Mass Spectrom.

[23] Chen Z, Glover MS, Li L (2018). Recent advances in ion mobility-mass spectrometry for improved structural characterization of glycans and glycoconjugates. Curr Opin Chem Biol.

[24] Gray CJ, Thomas B, Upton R, Migas LG, Eyers CE, Barran PE, et al. Applications of ion mobility mass spectrometry for high throughput, high resolution glycan analysis. Biochim Biophys Acta. 1860;2016:1688–709. 10.1016/j.bbagen.2016.02.003 26854953

[25] Mucha E, Stuckmann A, Marianski M, Struwe WB, Meijera G, Pagel K (2019). In-depth structural analysis of glycans in the gas phase. Chem Sci.

[26] Watanabe Y, Bowden TA, Wilson IA, Crispin M (1863). Exploitation of glycosylation in enveloped virus pathobiology. Biochim Biophys Acta Gen Subj.

[27] Hussain S, Miller JL, Harvey DJ, Gu Y, Rosenthal PB, Zitzmann N, et al. Strain-specific antiviral activity of iminosugars against human influenza A viruses. J Antimicrob Chemother. 2015;70:136–52. 10.1093/jac/dku349 PMC4267503 25223974

[28] Ritchie G, Harvey DJ, Stroeher U, Feldmann F, Feldmann H, Wahl-Jensen V, et al. Identification of *N*-glycans from Ebola virus glycoproteins by matrix-assisted laser desorption/ionisation time-of-flight and negative ion electrospray tandem mass spectrometry. Rapid Commun Mass Spectrom. 2010;24:571–85. 10.1002/rcm.4410 PMC3399782 20131323

[29] Ritchie G, Harvey DJ, Feldmann F, Stroeher U, Feldmann H, Royle L, et al. Identification of *N*-linked carbohydrates from severe acute respiratory syndrome (SARS) spike glycoprotein. Virology. 2010;399:257–69. 10.1016/j.virol.2009.12.020 PMC3412594 20129637

[30] Dunlop DC, Bonomelli C, Mansab F, Vasiljevic S, Doores KJ, Wormald MR, et al. Polysaccharide mimicry of the epitope of the broadly neutralizing anti-HIV antibody, 2G12, induces enhanced antibody responses to self oligomannose glycans. Glycobiology. 2010;20:812–23. 10.1093/glycob/cwq020 PMC2900896 20181792

[31] Harvey DJ, Sobott F, Crispin M, Wrobel A, Bonomelli C, Vasiljevic S, et al. Ion mobility mass spectrometry for extracting spectra of *N*-glycans directly from incubation mixtures following glycan release: application to glycans from engineered glycoforms of intact, folded HIV gp120. J Am Soc Mass Spectrom. 2011;22:568–81. 10.1007/s13361-010-0053-0 21472575

[32] Pritchard LK, Harvey DJ, Bonomelli C, Crispin M, Doores K, J. Cell- and protein-directed glycosylation of native cleaved HIV-1 envelope. J Virol 2015;89:8932–8944. 10.1128/JVI.01190-15 PMC4524065 26085151

[33] Behrens A-J, Vasiljevic S, Pritchard LK, Harvey DJ, Andev RS, Krumm SA, et al. Composition and antigenic effects of individual glycan sites of the trimeric HIV-1 envelope. Cell Rep. 2016;14:2695–706. 10.1016/j.celrep.2016.02.058 PMC4805854 26972002

[34] Behrens A-J, Harvey DJ, Milne E, Cupo A, Kumar A, Zitzmann N, et al. Molecular architecture of the cleavage-dependent mannose patch on a soluble HIV-1 envelope glycoprotein trimer. J Virol. 2017;91:e01894–16. 10.1128/JVI.01894-16 PMC5215339 27807235

[35] Behrens A-J, Kumar A, Medina-Ramirez M, Cupo A, Marshall K, Portillo VMC, et al. Integrity of glycosylation processing of a glycan-depleted trimeric HIV-1 immunogen targeting key B-cell lineages. J Proteome Res. 2018;17:987–99. 10.1021/acs.jproteome.7b00639 PMC5846105 29420040

[36] Struwe WB, Chertova E, Allen JD, Seabright GE, Watanabe Y, Harvey DJ, et al. Site-specific glycosylation of virion-derived HIV-1 env is mimicked by a soluble trimeric immunogen. Cell Rep. 2018;24:1958–66. 10.1016/j.celrep.2018.07.080 PMC6113929 30134158

[37] Seabright GE, Cottrell CA, van Gils MJ, D'addabbo A, Harvey DJ, Behrens A-J, et al. Networks of HIV-1 envelope glycans maintain antibody epitopes in the face of glycan additions and deletions. Structure. 2020;28:897–909. 10.1016/j.str.2020.04.022 PMC7416112 32433992

[38] Bowden TA, Crispin M, Harvey DJ, Aricescu AR, Grimes JM, Jones EY, et al. Crystal structure and carbohydrate analysis of Nipah virus attachment glycoprotein: a template for antiviral and vaccine design. J Virol. 2008;82:11628–36. 10.1128/JVI.01344-08 PMC2583688 18815311

[39] Bowden TA, Crispin M, Harvey DJ, Jones EY, Stuart DI (2010). Dimeric architecture of the Hendra virus attachment glycoprotein: evidence for a conserved mode of assembly. J Virol.

[40] Bowden TA, Crispin M, Graham SC, Harvey DJ, Grimes JM, Jones EY, et al. Unusual molecular architecture of the Machupo virus attachment glycoprotein. J Virol. 2009;83:8259–65. 10.1128/JVI.00761-09 PMC2715760 19494008

[41] Montesino R, Toledo JR, Sánchez O, Zamora Y, Barrera M, Royle L, et al. *N*-glycosylation pattern of E2 glycoprotein from classical swine fever virus. J Proteome Res. 2009;8:546–55. 10.1021/pr800725v 19093875

[42] Watanabe Y, Raghwani J, Allen JD, Seabright GE, Li S, Moser F, et al. Structure of the Lassa virus glycan shield provides a model for immunological resistance. Proc Natl Acad Sci U S A. 2018;115:7320–5. 10.1073/pnas.1803990115 PMC6048489 29941589

[43] Crispin M, Harvey DJ, Bitto D, Halldorsson S, Bonomelli C, Edgeworth M, et al. Uukuniemi phlebovirus assembly and secretion leave a functional imprint on the virion glycome. J Virol. 2014;88:10244–51. 10.1128/JVI.01662-14 PMC4136308 24942574

[44] Crispin M, Harvey DJ, Bitto D, Bonomelli C, Edgeworth M, Scrivens JH, et al. Structural plasticity of the Semliki Forest virus glycome upon interspecies transmission. J Proteome Res. 2014;13:1702–12. 10.1021/pr401162k PMC4428802 24467287

[45] Küster B, Wheeler SF, Hunter AP, Dwek RA, Harvey DJ (1997). Sequencing of *N*-linked oligosaccharides directly from protein gels: in-gel deglycosylation followed by matrix-assisted laser desorption/ionization mass spectrometry and normal-phase high performance liquid chromatography. Anal Biochem.

[46] Guile GR, Harvey DJ, O'Donnell N, Powell AK, Hunter AP, Zamze S, et al. Identification of highly fucosylated *N*-linked oligosaccharides from the human parotid gland. Eur J Biochem. 1998;258:623–56. 10.1046/j.1432-1327.1998.2580623.x 9874230

[47] Börnsen KO, Mohr MD, Widmer HM (1995). Ion exchange and purification of carbohydrates on a Nafion^(R)^ membrane as a new sample pretreatment for matrix-assisted laser desorption-ionization mass spectrometry. Rapid Commun Mass Spectrom.

[48] Giles K, Pringle SD, Worthington KR, Little D, Wildgoose JL, Bateman RH (2004). Applications of a travelling wave-based radio-frequency-only stacked ring ion guide. Rapid Commun Mass Spectrom.

[49] Hernández H, Robinson CV (2007). Determining the stoichiometry and interactions of macromolecular assemblies from mass spectrometry. Nat Protoc.

[50] Domon B, Costello CE (1988). A systematic nomenclature for carbohydrate fragmentations in FAB-MS/MS spectra of glycoconjugates. Glycoconj J.

[51] Bitto D, Harvey DJ, Halldorsson S, Doores KJ, Huiskonen JT, Bowden TA, et al. Determination of *N*-linked glycosylation in viral glycoproteins by negative ion mass spectrometry and ion mobility. Methods Mol Biol. 2015;1331:93–121. 10.1007/978-1-4939-2874-3_7 PMC4817836 26169737

[52] Harvey DJ (2005). Fragmentation of negative ions from carbohydrates: part 2, fragmentation of high-mannose *N*-linked glycans. J Am Soc Mass Spectrom.

[53] Harvey DJ (2005). Fragmentation of negative ions from carbohydrates: part 1; use of nitrate and other anionic adducts for the production of negative ion electrospray spectra from *N*-linked carbohydrates. J Am Soc Mass Spectrom.

[54] Harvey DJ (2005). Fragmentation of negative ions from carbohydrates: part 3, fragmentation of hybrid and complex *N*-linked glycans. J Am Soc Mass Spectrom.

[55] Harvey DJ, Royle L, Radcliffe CM, Rudd PM, Dwek RA (2008). Structural and quantitative analysis of *N*-linked glycans by MALDI and negative ion nanospray mass spectrometry. Anal Biochem.

[56] Rashid AM, Saalbach G, Bornemann S (2014). Discrimination of large maltooligosaccharides from isobaric dextran and pullulan using ion mobility mass spectrometry. Rapid Commun Mass Spectrom.

[57] Zhu F, Trinidad JC, Clemmer DE (2015). Glycopeptide site heterogeneity and structural diversity determined by combined lectin affinity chromatography/IMS/CID/MS techniques. J Am Soc Mass Spectrom.

[58] Gillece-Castro BL, Prakobphol A, Burlingame AL, Leffler H, Fisher SJ (1991). Structure and bacterial receptor activity of a human salivary proline-rich glycoprotein. J Biol Chem.

[59] Holten-Andersen L, Thaysen-Andersen M, Jensen SB, Buchwald C, Højrup P, Offenberg H, et al. Salivary tissue inhibitor of metalloproteinases-1 localization and glycosylation profile analysis. Acta Path Micro Im. 2011;119:741–9. 10.1111/j.1600-0463.2011.02796.x 21995626

[60] Harvey DJ, Crispin M, Scanlan C, Singer BB, Lucka L, Chang VT, et al. Differentiation between isomeric triantennary *N*-linked glycans by negative ion tandem mass spectrometry and confirmation of glycans containing galactose attached to the bisecting (β1-4-GlcNAc) residue in *N*-glycans from IgG. Rapid Commun Mass Spectrom. 2008;22:1047–52. 10.1002/rcm.3470 18327885

[61] Harvey DJ, Scarff CA, Edgeworth M, Struwe WB, Pagel K, Thalassinos K, et al. Travelling-wave ion mobility and negative ion fragmentation of high mannose *N*-glycans. J Mass Spectrom. 2016;51:219–35. 10.1002/jms.3738 PMC4821469 26956389

[62] Harvey DJ, Abrahams JL (2016). Fragmentation and ion mobility properties of negative ions from *N*-linked carbohydrates: part 7: reduced glycans. Rapid Commun Mass Spectrom.

[63] Tjondro HC, Ugonotti J, Kawahara R, Chatterjee S, Lokec I, Chen S, et al. Hyper-truncated Asn355- and Asn391-glycans modulate the activity of neutrophil granule myeloperoxidase. J Biol Chem. 2021;296:100144. 10.1074/jbc.RA120.016342 PMC7857493 33273015

[64] Ni W, Bones J, Karger BL (2013). In-depth characterization of *N*-linked oligosaccharides using fluoride-mediated negative ion microfluidic chip LC-MS. Anal Chem.

[65] Xie Y, Mota LM, Bergin A, O’Flaherty R, Jones A, Morgan B, et al. High-throughput and high-sensitivity *N*-glycan profiling: a platform for biopharmaceutical development and disease biomarker discovery. Anal Biochem. 2021;623:114205. 10.1016/j.ab.2021.114205 33891963

[66] Anumula KR (1993). Quantitative monosaccharide analysis of glycoproteins as anthranilyl derivatives by reverse phase HPLC. Glycobiology.

[67] Zhu Y, Liu X, Zhang Y, Wang Z, Lasanajak Y, Song X (2018). Anthranilic acid as a versatile fluorescent tag and linker for functional glycomics. Bioconjug Chem.

[68] Harvey DJ (2005). Collision-induced fragmentation of negative ions from *N*-linked glycans derivatized with 2-aminobenzoic acid. J Mass Spectrom.

